# Comparisons of lung and gluteus transcriptome profiles between yaks at different ages

**DOI:** 10.1038/s41598-019-50618-x

**Published:** 2019-10-02

**Authors:** Jin-Wei Xin, Zhi-Xin Chai, Cheng-Fu Zhang, Qiang Zhang, Yong Zhu, Han-Wen Cao, Qiu-Mei Ji, Jin-Cheng Zhong

**Affiliations:** 1 State Key Laboratory of Hulless Barley and Yak Germplasm Resources and Genetic Improvement, Lhasa, P.R. China; 2 Institute of Animal Science and Veterinary, Tibet Academy of Agricultural and Animal Husbandry Sciences, Lhasa, P.R. China; 3 Key Laboratory of Qinghai-Tibetan Plateau Animal Genetic Resource Reservation and Utilization, Sichuan Province and Ministry of Education, Southwest Minzu University, Chengdu, P.R. China

**Keywords:** Gene expression, Animal physiology, Molecular medicine

## Abstract

The yak, *Bos grunniens*, is the only large mammal in the Qinghai-Tibet Plateau and has been bred to provide meat, milk, and transportation. Previous studies indicate that the immune system contributes to the yak’s adaptation to high-altitude environments. In order to further investigate changes in immune function during yak development, we compared the transcriptome profiles of gluteus and lung tissues among yaks at 6, 30, 60, and 90 months of age. Analyses of significantly differentially expressed genes (DEGs) in lung tissues revealed that immune function was more activated at 6-months and less activated at 90-months than in the 30 and 60-month-old animals. DEG exploration in gluteal tissues revealed that immune functions were more highly activated at both 6 and 90-months, compared with 30 and 60-months. Immune system activation in the muscle and lung tissues of 30-month-old yaks may increase their resistance to infections, while decreased may be due to aging. Furthermore, the higher immune activation status in the gluteal tissues in 90-month-old yaks could be due to muscle injury and subsequent regeneration, which is supported by the fact that 5 unigenes related with muscle injury and 3 related to muscle regeneration displayed greater expression levels at 90-months than at 30 and 60-months. Overall, the present study highlights the important role of the immune system in yak development, which will facilitate future investigations.

## Introduction

The yak *Bos grunniens* is an amazing species, displaying a high tolerance to low oxygen and low temperature conditions^1^. Compared with related species, yaks have developed powerful respiratory and circulatory systems^2–4^, as well as special mechanisms underlying regulating the metabolism of nitrogen in the kidney^5,6^. Molecular studies have revealed upregulated expression of genes that might contribute to this adaptation to high altitude environments, including genes involved in hemocytogenesis, angiogenesis, heme binding, glycerolipid biosynthesis, electron carrier activity, immunity, cytochrome oxidase activity, and muscle proliferation and contractility^7–9^.

As the only large mammal species living in the Qinghai-Tibet Plateau, yaks provide meat and milk to local residents, while also serving agricultural and transportation roles^1^. To develop better breeding practices, more knowledge on the development and growth of yaks is required. As previously reported, yak dermis thickness increases with age, which may increase tolerance to low temperature^10^. Adult yaks had thinner media muscles in their pulmonary arteries and a thinner blood-air barrier in the lungs than did juvenile yaks^11^, which would improve the animal’s air-exchange efficiency. As age increases, functional thymic tissue is replaced by adipose, and connective tissues and thymic capsules thicken^12^, suggesting the changes in the immune system. These studies have explored morphological and histological changes during yak development. Few studies have been performed at a molecular level, however. One proteomics study revealed that proteins associated with cell adhesion, cell motility, keratinocyte differentiation, cytoskeleton organization, osteoblast differentiation, and fatty acid metabolism regulated horn development^13^. mRNA and protein expression levels of CD3 and S100β in the thymus were shown to decrease with age, whereas the expression level of caspase-3 increased with age, suggesting changes in immune function with advancing aging^12^. Obviously, the molecular understanding of yak development is still lacking, which is important for optimization of yak breeding practices.

Transcriptome sequencing is a powerful tool to screen changes in gene expression profiles, and has been widely used to investigate animal development^14,15^. Our previous study^16^ compared the transcriptome profiles of lung and gluteal tissues between yak, Tibetan cattle (high-altitude animals), Sanjiang cattle, and Holstein cattle (low-altitude animals). The results showed that 11 KEGG pathways associated with innate immunity were more activated in yak and Tibetan cattle than in low-altitude cattle breeds, suggesting that immune function might be important for yak adaptation to high-altitude environments. In the present study, in order to further explore changes in immune function during yak development, gluteal and lung tissues were collected from yaks of different ages and then subjected to transcriptome sequencing. Bioinformatics analyses were carried out to identify differentially expressed genes (DEGs) and enriched pathways and real-time quantitative PCR (RT-qPCR) was applied to validate gene expression levels. The potential roles of DEGs are discussed, and our results contribute to the current understanding of the molecular mechanisms underlying immune function development in the yak.

## Materials and Methods

### Ethics statement

The protocol used in the present study was approved by the Institutional Animal Care and Use Committee of Southwest Minzu University (Chengdu, P. R China) and all methods were carried out in accordance with the approved guidelines. No local regulations or laws were overlooked throughout the course of the investigation. Samples used in the present study were purchased from local farmers.

### Sample collection

In yak farms, breeders generally divide yaks into four age stages: juvenile (<1 year), youth (1–3 years), prime (3–7 years) and senior (>7 years). Yaks older than 7 years are generally sacrificed for meat. Thus, in the present study, samples from juvenile (6-month old), youth (30-month old), adult (60-month old), and senior (90-month old) yaks were collected for analyses. All yaks used in this study were raised at a local farm at Keqiong Village, Kamaduo Town, Leiwuqi County, Changdu City, P. R. China (96°22′45.26″N, 31°5′54.6″E, altitude = 4,343 m). Farmers regularly kill yaks to sell meat in the market. Between Oct 21^st^ and 22^nd^, 2017, lung and gluteal tissues were collected from healthy female animals at each stage (6 months, 30 months, 60 months, and 90 months), sacrificed at the local farm (altitude 4,343 m). Tissues were immediately frozen in liquid nitrogen. Three individuals were prepared for each age group as three biological replicates.

### RNA extraction and transcriptome sequencing

Total RNA was extracted using Biozol reagent (Bioer, Hangzhou, China), and was then visualized on 1% agarose gel. The RNA quality was estimated using a NanoPhotometer^®^ spectrophotometer (IMPLEN, CA, USA) and the Agilent Bioanalyzer 2100 system (Agilent Technologies, CA, USA). RNA samples with an RNA integrity number (RIN) higher than 8.0 were qualified. The RNA quantity was determined using the Qubit^®^ RNA assay kit (Life Technologies, CA, USA).

In order to construct sequencing libraries, mRNA was enriched by treating total RNA with the Epicentre Ribo-zeroTM rRNA removal kit (Epicentre, USA). Sequencing libraries were constructed using NEBNext^®^ Ultra^TM^ directional RNA library prep kit for Illumina^®^ (NEB, USA), according to the manufacturer’s instructions. Next, DNA fragments were purified with AMPure XP (Beckman Coulter, Beverly, USA) and treated with 3 μl of USER enzyme (NEB, USA) at 37 °C for 15 min. DNA fragments were then amplified using Phusion High-Fidelity DNA polymerase, universal PCR primers, and index (X) primers. Finally, PCR products were purified using AMPure XP and their quality was evaluated using the Agilent Bioanalyzer 2100.

Index-coded samples were clustered on a cBot cluster generation system with a HiSeq. 4000 PE cluster kit (Illumina). Afterwards, DNA libraries were sequenced on the Illumina Hiseq 4000 platform.

### Bioinformatics analyses

Clean reads were obtained from the raw data after removing adaptors, reads with an N ratio higher than 1%, and low quality reads (with >50% bases having Phred quality score ≤15). Afterwards, clean reads were mapped to the reference genome (BioProject number in GenBank: PRJNA435474) using STAR 2.51.b^17^. HTSeq v0.6.0^18^ was employed to calculate FPKM values (expected number of fragments per kilobase of transcript sequence per million base pairs sequenced) of each unigene. The relative expression levels of each gene were compared among different age groups using the DESeq2^19^ in R (https://www.r-project.org). Pairwise comparisons were performed for each unigene between each two groups (e.g., juvenile vs. youth, juvenile vs. senior adult, etc.). Comparisons with Q values <0.05 and |log_2_FoldChange >1 were considered significantly differentially expressed.

Differentially expressed genes (DEGs) were mapped to the KEGG database (Kyoto Encyclopedia of Genes and Genomes) of *Bos mutus* (wild yak, database ID: T02919 in https://www.genome.jp) using ClusterProfiler3^20^ to find out significantly enriched pathways. P values were adjusted using the BH method^21^. To prevent a high false discovery rate (FDR) in multiple testing, Q values were also estimated for FDR control^22^. The cut-off of adjusted P values and Q values was both set at 0.05.

### Real-time quantitative PCR

To verify the expression levels of DEGs calculated by FPKM, qPCR was performed. cDNA was synthesized from total RNA (the same RNA samples for Illumina sequencing) using a BioRT cDNA first strand synthesis kit (Bioer, Hangzhou, China) with the oligo(dT) primer. qPCR reactions were performed on a Line Gene9600 Plus qPCR machine (Bioer, Hangzhou, China) using BioEasy master mix (Bioer, Hangzhou, China). Each reaction was repeated three times. Three biological replicates were also performed for each age group. Primers used in the present study are listed in Supplementary Table S1. Glyceraldehyde phosphate dehydrogenase (GAPDH) was used as the internal control^16,23^. The relative expression level of each gene was calculated using the 2^−ΔΔCt^ method^24^. Student’s t tests were performed to compare the relative expression level of each unigene between each two age groups. P < 0.05 was considered statistically significant.

## Results and Discussion

### Illumina sequencing

The original sequencing files have been deposited in the National Center for Biotechnology Information (NCBI) database (bioproject number PRJNA512958). A total of 12 lung and 12 gluteus samples were successfully sequenced, generating 78.7 M to 101.0 M clean reads. The sequence data of every sample showed Q20 values higher than 96.7% and Q30 values higher than 92.0%, suggesting that the results of transcriptome sequencing were technically qualified (Supplementary Table S2).

### Differentially expressed genes

When the transcriptome profiles of the 30- and 60-month old yaks were compared, only one unigene was differentially expressed in gluteal tissues (Q < 0.05; Foldchange > 2) and no unigenes showed significant differences in lung tissues (Table 1).

**Table 1:** Numbers of differentially expressed genes in gluteal and lung tissues of 6, 30, 60, and 90-month old yaks.

	6-month	30-month	60-month	90-month
6-month	—	238	338	248
30-month	219	—	1	253
60-month	115	0	—	374
90-month	35	21	13	—

Blow diagonal: lung tissue; Above diagonal: gluteus.

Compared with 6-month old yaks, 219 and 115 DEGs in gluteal tissues and 238 and 338 DEGs in lung tissues were detected in 60- and 90-month old yaks, respectively (Q < 0.05; Foldchange > 2). Compared with 90-month old yaks, 21 and 13 DEGs in gluteal tissues and 253 and 374 DEGs in lung tissues were detected in 30- and 60-month old yaks, respectively (Q < 0.05; Foldchange > 2; Table 1).

**Figure 1:**
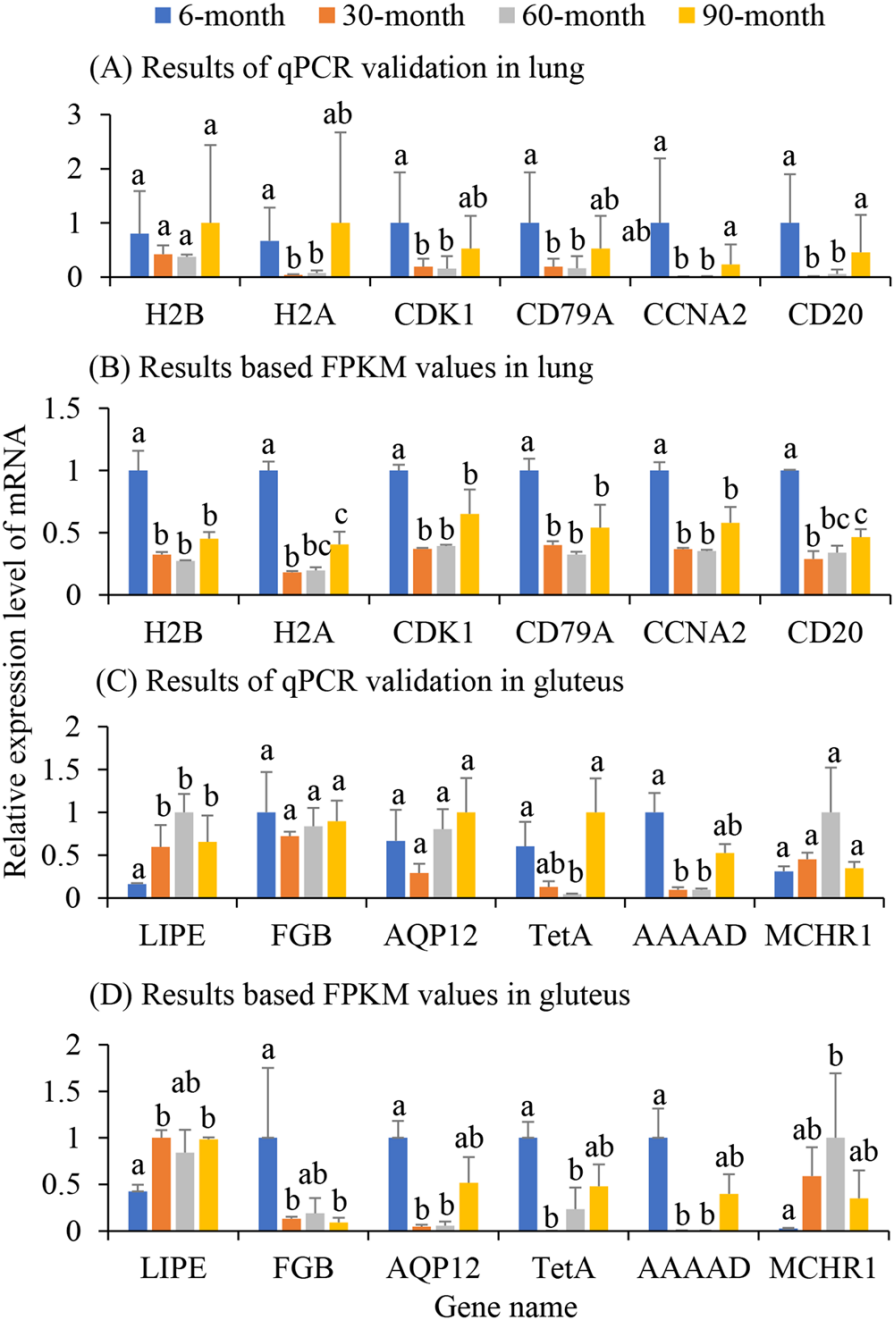
Real-time quantitative PCR validation of differentially expressed genes. H2B: histone H2B; H2A: histone H2A; CDK1: cyclin-dependent kinase 1; CD79A: CD79A antigen; CCNA2: cyclin (**A**) CD20: B-lymphocyte antigen CD20; LIPE: Hormone-sensitive lipase; FGB: Fibrinogen; AQP12: Aquaporin 12; TetA: Tetracycline resistance protein; AAAAD: AAA+ ATPase domain; MCHR1: Melanin-concentrating hormone 1. Student’s t-tests were performed to determine differences in each unigene between age groups. Different letters above the bars indicated significant differences.

Six unigenes from the lung tissues and six from gluteal tissues were selected for qPCR to validate the expression levels calculated by FPKM values. Melting curves showed a single peak for all tested genes, suggesting that the PCR primers were specific. In the lung tissue five of the six unigenes tested were identified as being differentially expressed between the 6-month yaks and other age groups in the transcriptome analysis, using qPCR (Student’s t-test, P < 0.05), while one (histone H2B) was not (Student’s t-test, P > 0.05). In the gluteal tissues, four of the six tested unigenes revealed similar changing trends among the four age groups between qPCR validation and Illumina sequencing (Pearson’s correlation, P < 0.05). The other two, aquaporin 12 (AQP12) and fibrinogen (FGB), displayed significant differences between 3- and 30-month old yaks (Student’s t-test, P < 0.05) in the transcriptome analyses but no significant change (Student’s t-test, P > 0.05) in the qPCR validation (Fig. 1). Overall, these results suggest that the transcriptome results are reliable.

### Comparison of lung tissues between yaks of different ages

DEGs in lung tissues comparing 6-month and 30-month yaks, as well as 6-month and 60-month yaks, were separately subjected to KEGG enrichment analyses. The results were quite similar and were combined in one list, generating 34 KEGG pathways (Supplementary Table S3). Among the results, “ko05414 dilated cardiomyopathy” might respond to low oxygen conditions. Results related to immune function include “ko05340 primary immunodeficiency”, “ko04662 B cell receptor signaling pathway”, and “Ko05330 allograft rejection”. Moreover, several typical regulating pathways (“ko04024 cAMP signaling pathway”, “ko04064 NF-kappa B signaling pathway”, and “ko04115 p53 signaling pathway”) were also detected, which might function in signal transduction. Concurrently, comparisons between 30- and 90-month old yaks and between 60- and 90-month yaks were significantly enriched in three KEGG pathways, including “ko05322 systemic lupus erythematosus”, “ko05034 alcoholism” and “ko05152 tuberculosis” (Supplementary Table S3).

**Figure 2:**
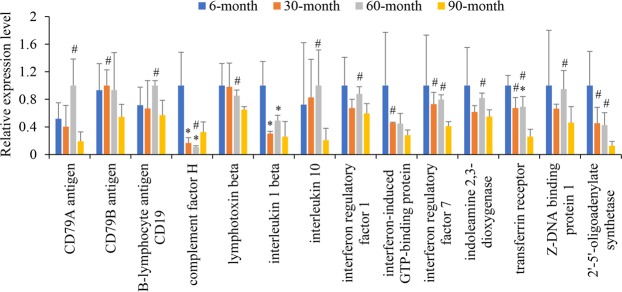
Relative expression levels of differentially expressed genes associated with immune function in lung tissue. All data were calculated based on the FPKM values and were normalized by defining the highest FPKM value among the four age groups as one. Errors donate standard deviation. Student’s t-tests were performed to determine differences in each unigene between age groups. *Significantly different from 6-month old group (P < 0.05). ^#^Significantly different from 90-month (P < 0.05).

To further explore changes in immune function during yak development, DEGs in immunity-related KEGG pathways were picked up. B-lymphocyte CD19 and CD79A antigens were significantly lower in 90-month yak growth compared with 60-month yaks (Student’s t-test, P < 0.05), with change folds of 1.75 and 5.2, respectively. CD79B antigen was significantly lower in the 90-month group than in the 30-month group (Student’s t-test, P < 0.05; 1.83 times; Fig. 2 and Supplementary Table S4). CD79a and CD79b antigens are components of the cell-surface B-cell receptor (BCR) and CD19 is a B cell surface molecule modifying signals generated through BCR. CD79A, CD79B, and CD19 are involved in recognition and subsequent signal transduction of pathogens and other foreign substances in B cells^25,26^. Downregulation of CD79A, CD79B, and CD19 in 90-month old yaks suggests that the B-cell function declined at 90 months compared to 30- and 60-month animals.

Transferrin receptor expression is upregulated during T cell activation^27^, which is required for DNA synthesis and cell division^28^. In the present study, transferrin receptor levels were 2.59 and 2.67 times significantly lower in 90-month old yaks than in 30- and 60-month old yaks, respectively (Student’s t-test, P < 0.05, Fig. 2 and Supplementary Table S4), suggesting that activated T cell levels might be reduced in 90-month old yaks, similar to those of B-cells.

Development, differentiation, and T and B lymphocyte function can be mediated by cytokines, such as complement factor H (CFH) not only plays a critical role in the homeostasis of the complement system in plasma but also functions as an important regulatory protein in innate immune system^29^. Compared with the 30- and 60-month old groups, expression levels of CFH were 6.03 and 9.32 times significantly higher, and those of the transferrin receptor were 1.49 and 1.45 times significantly higher, at the 6-month stage, respectively (Student’s t-test, all P values < 0.05; Fig. 2 and Supplementary Table S4). Increased CFH and transferrin receptor expression levels suggested that immune functions might be more activated at 6-month stage compared with 30-month and 60-month stages. Moreover, interleukin-1 (IL-1) could induce production of CFH^30^ and transferrin receptor^31^. In the present study, expression level of IL-1 was 3.31 and 2.04 times significantly higher at 6-month stage in comparison to 30-month and 60-month stage, respectively (Student’s t-test, both P values < 0.05), displaying similar changing trends with CFH and transferrin receptor (Pearson’s correlation, P < 0.05; Fig. 2 and Supplementary Table S4). These results suggested that higher activation status of immune function in 6-month yak might be regulated by IL-1. Interferons (IFNs) are mainly synthesized and released by helper CD4 T lymphocytes, monocytes, macrophages, and endothelial cells^32^. These cytokines regulate immune and inflammatory response to infections^33,34^. In the present study, IFN regulatory factor (IRF) 1 levels were 1.5 times significantly lower in 90-month old yaks than in 60-month old animals (Student’s t-test, P < 0.05). IRF-7 levels were 1.77 and 1.93 times significantly lower in 90-month-olds than in 30- and 60-month-olds, respectively (Student’s t-test, both P < 0.05). IFN-induced GTP-binding protein was 1.64 times significantly lower in 90-month old yaks than in 30-month yaks (Student’s t-test, P < 0.05). Moreover, 2′-5′-oligoadenylate synthetase (OAS2), which is stimulated by IFN^35^, also displayed significantly lower levels in the 90-month group than in the with 30- and 60-month groups, with change folds of 3.58 and 3.38, respectively (Student’s t-test, all P < 0.05). An upstream activator of IRF, Z-DNA binding protein 1 (ZBP1), which can detect DNA from viral, bacterial, or even host origin^36^, was 1.31 times significantly lower in the 90-month yaks than in the 60-month yaks (Student’s t-test, P < 0.05; Fig. 2 and Supplementary Table S4). These results indicate that IFN levels might be reduced in 90-month old yaks compared with 30- and 60-month old yaks. The expression level of IL-10 was 4.83 times lower in the 90-month than in the 60-month group (Student’s t-test, P < 0.05). Furthermore, indoleamine 2,3-dioxygenase (IDO), which has been reported to enhance production of interleukin 10^37^, showed a 1.49 times significantly lower expression level at 90-months than at 60-months (Student’s t-test, P < 0.05; Fig. 2 and Supplementary Table S4). Changes in the levels of these two proteins indicate that IL-10 is less expressed in 90-month old yaks compared to 60-month old yaks. In addition, the cytokine lymphotoxin showed significantly lower levels (1.31 times) in the 90-month than the 60-month group (Student’s t-test, P < 0.05). IFN regulates B-cell function in humans^38^ and IL-10^39^ and lymphotoxin^40^ act as autocrine growth or activation factors for B lymphocytes. Thus, it is possible that reduced B-cell function in 90-month old yaks is probably attributed to decreased levels of IFN, IL-10 and lymphotoxin.

Activation of immune functions in 6-month yaks might be attributed to their immature physiological development. First, high-altitude environments experience strong UV radiation, low oxygen, and low temperatures. These factors can negatively affect the immune system and make animals more susceptible to infections^41^. As reported previously, the dermis thickness is lower in juvenile yak than adults^10^. Thus, 6-month old yaks should be more susceptible to low oxygen, low temperature, and high UV exposure. Increased immune activation could help juvenile yaks resist the dangers of a high-altitude environment. Second, the lungs are weaker in juvenile yaks than in adults. Enhanced immune function in juveniles could prevent acute mountain sickness, high altitude pulmonary edema, and high altitude cerebral edema^42^.

**Figure 3:**
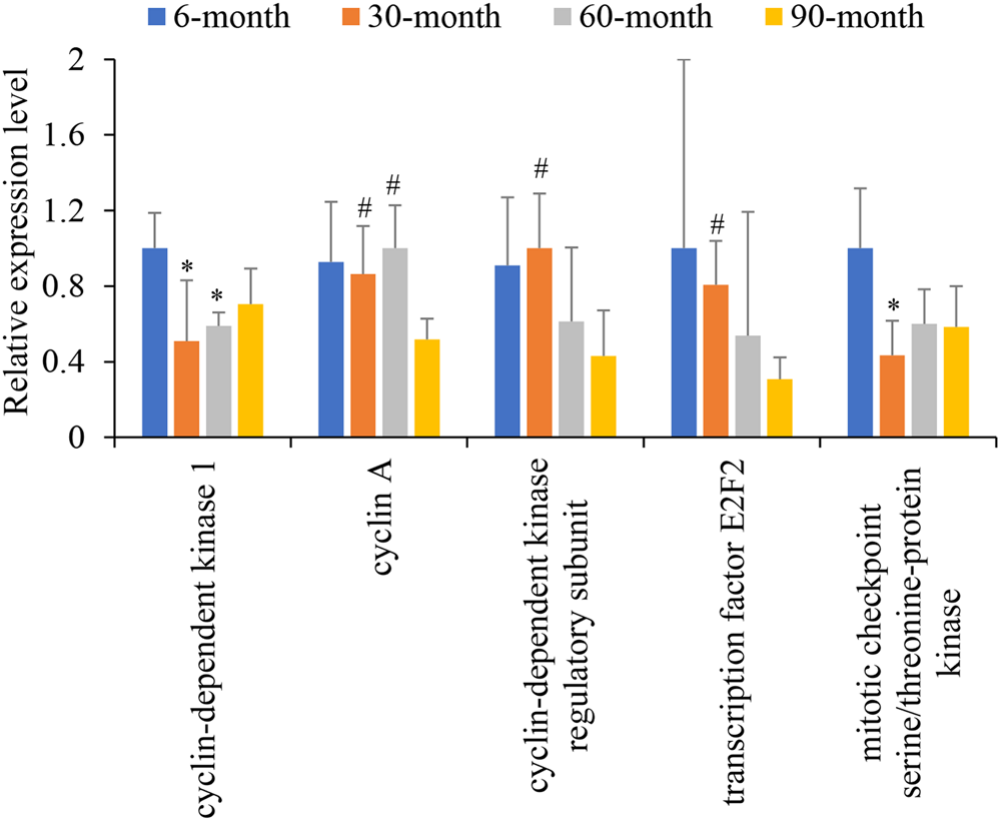
Relative expression levels of differentially expressed genes associated with the cell cycle in lung tissue. All data were calculated based on the FPKM values and were normalized by defining the highest FPKM value among the four age groups as one. Errors donate standard deviation. Student’s t-tests were performed to determine differences in each unigene between age groups. *Significantly different from 6-month old group (P < 0.05). ^#^Significantly different from 90-month old group (P < 0.05).

Moreover, unigenes related to the cell cycle are also differentially expressed in lung tissues among different aged yaks. Cyclins regulate the cell cycle by activating cyclin-dependent kinases (CDKs)^43^. Cyclin-dependent kinase regulatory subunit 1 (CKS1) regulates the cell cycle to increase cell numbers^44^. Mitotic checkpoint serine/threonine-protein kinase (Bub1) and condensin complex subunit 3 are required for chromosome congression, assembly, and segregation during mitosis and meiosis^45,46^. Overexpression of the transcription factor E2F2 in nonproliferating rabbit corneal endothelial cells has been shown to induce cell cycle progression without prompting significant apoptosis^47^. In the present study, CDK1 levels were 1.96 and 1.70 times significantly more highly expressed in 6-month old yaks compared with 30-month and 60-month, respectively (Student’s t-test, both P < 0.05). Cyclin A levels were 1.67 and 1.93 times significantly lower in 90-month old lung tissues than in those from 30- and 60-month animals, respectively (Student’s t-test, P < 0.05). CKS1 and E2F2 levels were 2.33 and 2.63 times significantly lower at 90-months than at 30-months (Student’s t-test, both P < 0.05). BUB1 showed 2.31 times significantly higher expression at 6-months than at 30-months (Student’s t-test, P < 0.05; Fig. 3 and Supplementary Table S4). These results suggest that the cell cycle was promoted at 6-months but was decreased at 90-months, in comparison with 30- and 60-month yak lung tissues. Thus, the growth of lung tissues should be the fastest at 6-month of age, following by 30- and 60-months, and reaching the slowest rate of growth at 90-months. Moreover, 12 DEGs encoding histone showed significantly higher expression levels in 6-month old yak lung tissues than in 30-month tissues (Student’s t-test, P < 0.05). Three histone unigenes were significantly lower at 90-months than at 60-months (Student’s t-test, P < 0.05; Fig. 3 and Supplementary Table S4). Histones are the chief protein component of chromatin, and must accumulate before cell division. The changes in histone expression levels also support our hypothesis in relation to cell cycle.

### Comparison of gluteal tissues between yaks of different ages

In gluteal tissues, DEGs comparisons between 6- and 30-months and between 6- and 60-months were separately subjected to KEGG enrichment analyses. The as-obtained pathways were combined into one list. Finally, a total of four KEGG pathways were significantly over-represented when comparing 6- and 30-month yaks, and between 6- and 60-month yaks. Comparisons between 30- and 90-month and between 60- and 90-month gluteal tissues showed 26 significantly enriched KEGG pathways. Among them, 14 were functionally related to the immune system, including “ko04650 natural killer cell mediated cytotoxicity”, “ko04672 intestinal immune network for IgA production”, “ko04060 cytokine-cytokine receptor interaction”, and “ko05340 primary immunodeficiency” (Supplementary Table S5).

**Figure 4:**
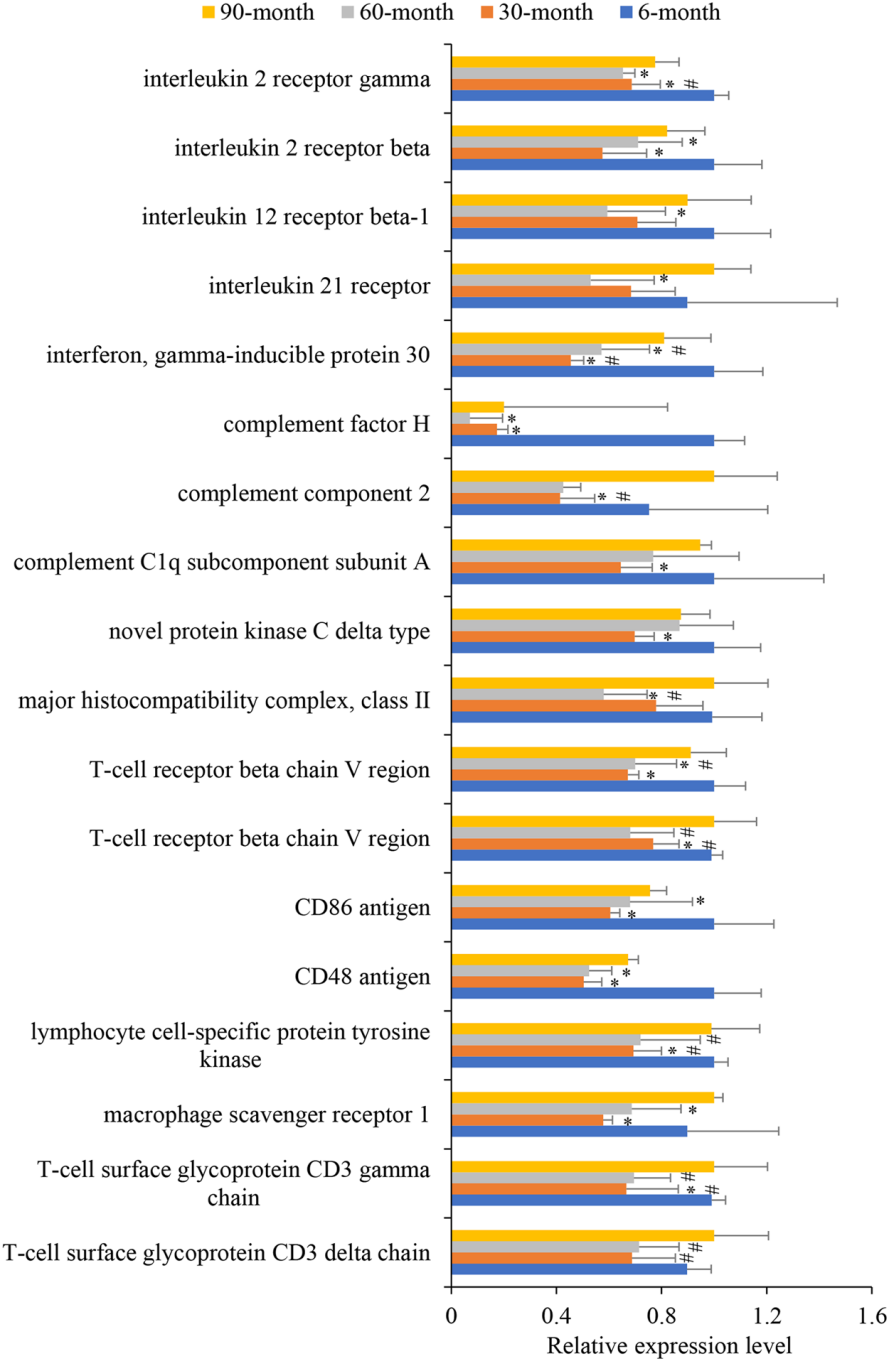
Relative expression levels of differentially expressed genes associated with immune function in gluteal tissue. All data were calculated based on the FPKM values and were normalized by defining the highest FPKM value among the four age groups as one. Errors donate standard deviation. Student’s t-tests were performed to determine differences in each unigene between age groups. *Significantly different from 6-month old group (P < 0.05). ^#^Significantly different from 90-month old group (P < 0.05).

Compared with the 6-month group, expression levels of macrophage scavenger receptor 1 were 1.55 and 1.31 times lower, CD48 levels were 1.98 and 1.91 times lower, CD86 levels were 1.65 and 1.47 times lower, T-cell receptor beta chain V region (BmuPB011165) levels were 1.49 and 1.43 times lower, complement factor H levels were 5.75 and 14.13 times lower, and interferon levels were 2.20 and 1.73 times lower than the same levels at 30- and 60-months, respectively (all the comparisons were statistically significant, Student’s t-test, P values < 0.05; Fig. 4 and Supplementary Table S6). These results indicate that immune function at 6-months is more activated than at 30- and 60-months, which could be due to the immature developmental status and increased need to resist high-altitude environments at 6-months of age, similar to the results observed in lung tissues. Moreover, interleukin 2 receptor (IL-2R) beta levels were 1.74 and 1.41 times significantly higher and IL-2R gamma levels were 1.46 and 1.53 times significantly higher at 6-months than at 30- and 60-months, respectively (Student’s t-test, P < 0.05). As reported, IL-2R is responsible for transducing signals from IL-2 and T-cell dependent activity^48^. Thus, it is possible that the activation of immune factors in 6-month old muscle tissues is regulated by IL-2.

Compared with 30- and 60-month yaks, the T-cell surface glycoprotein CD3 delta chain levels were 1.46 and 1.40 times higher, CD3 gamma chain levels were 1.50 and 1.44 times higher, lymphocyte cell-specific protein tyrosine kinase levels were 1.43 and 1.37 times higher, T-cell receptor beta chain V region (BmuPB011166) levels were 1.30 and 1.47 times higher, and interferon levels were 1.78 and 1.42 times higher at 90-months, respectively (all the comparisons were statistically significant, Student’s t-test, P values < 0.05). These results indicate that immune activation in muscle tissues is higher in 90-month old yaks than in 30- and 60-month animals. In older yaks, muscles might be more vulnerable and muscle injuries would induce immune activation^49^. To test this hypothesis, genes associated with muscle tenderness, injury, and regeneration were investigated. First, at the 90-month stage, four unigenes encoding collagen/elastin were significantly lower than those at the 30-month stage (change fold ranged from 1.63 to 2.01) and three collagen/elastin unigenes were significantly depressed compared with levels at 60-months (change fold ranged from 1.69 to 2.01) (Student’s t-test, all P values < 0.05; Fig. 4 and Supplementary Table S6). Muscle tenderness has been positively correlated with intramuscular collagen content in bovines^50^. Our results suggest that 90-month old yaks might suffer muscle injuries more readily, due to low muscle tenderness, than do 30- and 60-month old yaks. Second, four unigenes encoding the C-C chemokine receptor were significantly higher in 90-month than in 30-month yaks (change folds ranged from 1.54 to 1.82, Student’s t-test, all P values < 0.05), and the C-C chemokine receptor type 5 level was 1.41 times higher in 90-month than in 60-month old yaks (Student’s t-test, P < 0.05). The unigene BmuPB014660, encoding tumor necrosis factor receptor superfamily member 5, was 1.41 times significantly higher than in 60-month old yaks (Student’s t-test, P < 0.05). The expression level of integrin alpha M was 1.94 times significantly higher at 90-months than at 60-months (Student’s t-test, P < 0.05; Supplementary Table S6). C-C chemokine receptor^51,52^, tumor necrosis factor (TNF)^53,54^, and integrin^55^ have been reported to participate in muscle injury responses and the subsequent regeneration of new tissues. Thus, overexpression of these unigenes at 90-months of age suggests that 90-month old yaks might undergo more muscle injuries. Furthermore, the ras-related C3 botulinum toxin substrate 2 level was 1.29 times significantly higher in 90-month than 60-month old yaks (Student’s t-test, P < 0.05) The ras superfamily regulates cell differentiation, proliferation, and cytoskeletal organization^56^. The observed high expression of ras-related C3 botulinum toxin substrate 2 supports the evidence that increased regeneration of new muscle tissues might take place in 90-month old yaks. Overall, these results indicated that muscle tissue at 90-months of age was more highly activated than at 30- and 60-months of age, which is probably due to the lower muscle tenderness and more severe injuries at 90-months.

## Conclusions

The present study reveals that immune activation status differs between yaks of different ages. In lung tissues, immune function was more activated at 6-months of age and less activated at 90-months, compared with activation at 30- and 60-months. In gluteal tissues, immune function was more activated at 6- and 90-months than at 30- and 60-months. This activation of the immune system could increase the resistance of juvenile yaks to high-altitude environments. Enhanced immune function in senior yak gluteal tissues might be a response to muscle injuries resultant from low muscle tenderness.

## Data Availability

The original sequencing files have been deposited in the National Center for Biotechnology Information (NCBI) with the bioproject number PRJNA512958.

## Supplementary information

supplementary materials
