# Supplementary material for: Comparisons of lung and gluteus transcriptome profiles between yaks at different ages
Source: Sci Rep. 2019 Oct 2;9:14213. doi: 10.1038/s41598-019-50618-x (PMC6775228; doi:10.1038/s41598-019-50618-x)
Supplement: Supplementary file 1 — supplementary materials [file 41598_2019_50618_MOESM1_ESM.docx]

**Supplementary Materials:**

**Comparisons of lung and gluteus transcriptome profiles between yaks at different ages**

Jin-Wei Xin^1,2,#^, Zhi-Xin Chai^3,#^, Cheng-Fu Zhang^1,2^, Qiang Zhang^1,2^, Yong Zhu^1,2^, Han-Wen Cao^1,2^, Qiu-Mei Ji^1,2,*^, Jin-Cheng Zhong^3,*^

^1^. State Key Laboratory of Hulless Barley and Yak Germplasm Resources and Genetic Improvement, Lhasa, P. R. China

^2^. Institute of Animal Science and Veterinary, Tibet Academy of Agricultural and Animal Husbandry Sciences, Lhasa, P. R. China

^3^. Key Laboratory of Qinghai-Tibetan Plateau Animal Genetic Resource Reservation and Utilization, Sichuan Province and Ministry of Education, Southwest Minzu University, Chengdu, P. R. China

^#^ J-W Xin and Z-X Chai contributed equally to this work.

^*^ Corresponding author: Q-M Ji ([xinjinwei18@163.com)](mailto:xinjinwei18@163.com)) and J-C Zhong ([zhongjincheng518@126.com](mailto:zhongjincheng518@126.com))

**Supplementary Table S1. Primers.** The upper and lower sequences are the forward and reverse primers, respectively.

| Unigene ID and gene name | Primer sequence |
| --- | --- |
| BmuPB016882, Hormone-sensitive lipase | CAAAATGAACCACCTGCCCA |
|  | GTGGCTGGTGAATCGATGTC |
| BmuPB017281, Fibrinogen | TGGGACCCACAAAGCTTTTG |
|  | ACCAGTTGAGAGGCTCCTTC |
| BmuPB021639, Aquaporin 12 | CCGAGTCTTTGGGTCAGTCT |
|  | CATTCGCCCAGTCTGTAAGC |
| BmuPB021749, Tetracycline resistance protein | CACAGAGCCCAGTGTTTTCC |
|  | GACACAAAGGCCACAACAGT |
| BmuPB022055, AAA+ ATPase domain | TATCGTCCACCACCACTCTG |
|  | GCTCCTGTTCTTCCCACGAT |
| BmuPB022068, Melanin-concentrating hormone 1 | TCTTGAGGAAGGCACCAGAG |
|  | AGTGCCTTGCGGTAGATCAT |
| BmuPB008322, histone H2B | TCTCATCTAAGGCCATGGGT |
|  | TAGTTACGGCCTTGGTACCC |
| BmuPB008346, histone H2A | CTGACGGCCGAGATCCTAG |
|  | AAACACCACCCTGAGCAATG |
| BmuPB013384, cyclin-dependent kinase 1 | ACGCATGAGGTAGTGACACT |
|  | AGCTTCCTGGTTTCCACTTG |
| BmuPB016862, CD79A antigen | AACAAGACTCACAGGGGCAT |
|  | ATGTTCTGCCATCGTTTCCG |
| BmuPB018681, cyclin A | CAGGACCAAGAAAGCCACTG |
|  | CCAAATGCAGGGTCTCGTTC |
| BmuPB019354, B-lymphocyte antigen CD20 | TGGGGAGGCATCATGTTCAT |
|  | GGTTAGCTCGCTCACAGTTG |

**Table S2. Illumina transcriptome sequencing.**

| Sample code | Age (months) | Total reads | Total bases | Q20 | Q30 |
| --- | --- | --- | --- | --- | --- |
| Gluteus | | | | | |
| 11 | 6 | 90,388,522 | 13,558,278,300 | 97.17% | 92.90% |
| 7 | 6 | 87,590,344 | 13,138,551,600 | 97.09% | 92.91% |
| 8 | 6 | 82,711,736 | 12,406,760,400 | 97.15% | 92.88% |
| 1 | 30 | 89,849,262 | 13,477,389,300 | 97.32% | 93.20% |
| 5 | 30 | 94,530,242 | 14,179,536,300 | 97.19% | 92.96% |
| 6 | 30 | 78,733,474 | 11,810,021,100 | 97.19% | 93.07% |
| 12 | 60 | 79,303,042 | 11,895,456,300 | 97.16% | 92.87% |
| 2 | 60 | 91,773,632 | 13,766,044,800 | 97.33% | 93.30% |
| 3 | 60 | 81,165,810 | 12,174,871,500 | 97.19% | 92.90% |
| 10 | 90 | 88,273,646 | 13,241,046,900 | 97.19% | 93.00% |
| 4 | 90 | 88,172,328 | 13,225,849,200 | 97.01% | 92.72% |
| 9 | 90 | 80,199,662 | 12,029,949,300 | 96.74% | 91.99% |
| Lung tissue | | | | | |
| 11 | 6 | 83,603,988 | 12,540,598,200 | 97.29% | 93.15% |
| 7 | 6 | 101,042,550 | 15,156,382,500 | 96.77% | 92.18% |
| 8 | 6 | 79,658,978 | 11,948,846,700 | 96.98% | 92.60% |
| 1 | 30 | 80,050,806 | 12,007,620,900 | 97.21% | 93.03% |
| 5 | 30 | 81,520,614 | 12,228,092,100 | 97.08% | 92.84% |
| 6 | 30 | 91,101,332 | 13,665,199,800 | 97.11% | 92.92% |
| 12 | 60 | 94,836,062 | 14,225,409,300 | 97.00% | 92.63% |
| 2 | 60 | 86,833,696 | 13,025,054,400 | 97.37% | 93.42% |
| 3 | 60 | 80,711,142 | 12,106,671,300 | 97.27% | 93.18% |
| 10 | 90 | 82,823,284 | 12,423,492,600 | 97.23% | 93.18% |
| 4 | 90 | 83,200,808 | 12,480,121,200 | 96.87% | 92.38% |
| 9 | 90 | 85,586,970 | 12,838,045,500 | 97.22% | 93.06% |

**Table S3. Comparison of significantly enriched KEGG pathways in lung tissues between yaks at different ages.**

| ID | Gene Ratio | Adjust P | Q value |
| --- | --- | --- | --- |
| Comparisons between 6 and 30/60-month old | | | |
| ko05322 Systemic lupus erythematosus | 28/96 | 0.000 | 0.000 |
| ko05034 Alcoholism | 22/96 | 0.000 | 0.000 |
| ko05143 African trypanosomiasis | 8/96 | 0.000 | 0.000 |
| ko05203 Viral carcinogenesis | 15/96 | 0.000 | 0.000 |
| ko02010 ABC transporters | 9/96 | 0.000 | 0.000 |
| ko04640 Hematopoietic cell lineage | 9/96 | 0.000 | 0.000 |
| ko05140 Leishmaniasis | 8/96 | 0.000 | 0.000 |
| ko04110 Cell cycle | 10/96 | 0.000 | 0.000 |
| ko04976 Bile secretion | 9/96 | 0.000 | 0.000 |
| ko05340 Primary immunodeficiency | 6/96 | 0.000 | 0.000 |
| ko04662 B cell receptor signaling pathway | 7/96 | 0.000 | 0.000 |
| ko05202 Transcriptional misregulation in cancer | 11/96 | 0.001 | 0.000 |
| ko05310 Asthma | 6/96 | 0.001 | 0.001 |
| ko05162 Measles | 9/96 | 0.003 | 0.002 |
| ko05146 Amoebiasis | 7/96 | 0.003 | 0.003 |
| ko04024 cAMP signaling pathway | 10/96 | 0.005 | 0.004 |
| Ko05330 Allograft rejection | 6/96 | 0.005 | 0.004 |
| ko05150 Staphylococcus aureus infection | 6/96 | 0.006 | 0.005 |
| ko04064 NF-kappa B signaling pathway | 7/96 | 0.007 | 0.005 |
| ko05323 Rheumatoid arthritis | 7/96 | 0.007 | 0.005 |
| ko05169 Epstein-Barr virus infection | 9/96 | 0.010 | 0.008 |
| ko04664 Fc epsilon RI signaling pathway | 5/96 | 0.012 | 0.009 |
| ko04111 Cell cycle - yeast | 5/96 | 0.012 | 0.009 |
| ko05152 Tuberculosis | 8/96 | 0.015 | 0.012 |
| ko04672 Intestinal immune network for IgA production | 5/96 | 0.019 | 0.015 |
| ko04145 Phagosome | 7/96 | 0.019 | 0.015 |
| ko04914 Progesterone-mediated oocyte maturation | 5/96 | 0.021 | 0.016 |
| ko04666 Fc gamma R-mediated phagocytosis | 5/96 | 0.025 | 0.019 |
| ko05320 Autoimmune thyroid disease | 5/96 | 0.037 | 0.028 |
| ko05414 Dilated cardiomyopathy | 5/96 | 0.037 | 0.028 |
| ko04623 Cytosolic DNA-sensing pathway | 4/96 | 0.039 | 0.030 |
| ko04115 p53 signaling pathway | 4/96 | 0.040 | 0.030 |
| ko04650 Natural killer cell mediated cytotoxicity | 6/96 | 0.044 | 0.034 |
| ko05133 Pertussis | 4/96 | 0.050 | 0.038 |
| Comparisons between 30/60-month and 90-month old | | | |
| ko05322 Systemic lupus erythematosus | 3/7 | 0.003 | 0.002 |
| ko05034 Alcoholism | 3/7 | 0.003 | 0.002 |
| ko05152 Tuberculosis | 2/7 | 0.043 | 0.029 |

**Table S4. FPKM values of differentially expressed genes in yak lung tissues at different ages (mean ± SD).**

| ID and name of unigene | 6-month | 30-month | 60-month | 90-month |
| --- | --- | --- | --- | --- |
| Cell cycle-related genes | | | | |
| BmuPB020811, histone H4 | 16.75±8.04 | 5.29±1.83^*^ | 8.71±2.25 | 5.65±2.49 |
| BmuPB008345, histone H2B | 45.48±12.03 | 19.21±2.62^*^ | 29.43±7.44 | 19.90±2.61 |
| BmuPB020809, histone H3 | 13.80±3.43 | 4.89±2.14^*^ | 5.25±1.60^*^ | 4.82±0.80 |
| BmuPB008320, histone H3 | 19.21±7.62 | 6.82±4.19^*^ | 8.82±0.96^*^ | 7.24±3.25 |
| BmuPB008344, histone H2A | 4.76±2.40 | 1.33±0.90^*^ | 1.70±0.60^*^ | 0.88±0.57 |
| BmuPB022427, histone H2A | 14.90±10.80 | 1.42±1.03^*#^ | 1.52±1.03^*^ | 3.62±1.41 |
| BmuPB020812, histone H2B | 18.51±10.18 | 8.07±2.53 | 9.83±0.75^#^ | 5.90±1.89 |
| BmuPB008331, histone H4 | 37.97±4.30 | 20.40±8.66^*^ | 25.14±3.48^*^ | 24.80±8.54 |
| BmuPB008321, histone H2A | 43.34±10.36 | 27.86±4.29^*^ | 43.12±6.63 | 43.56±13.88 |
| BmuPB022428, histone H4 | 4.42±1.62 | 1.38±0.91^*^ | 1.23±1.11^*^ | 0.82±0.85 |
| BmuPB020810, histone H4 | 112.63±37.34 | 31.69±6.27^*^ | 43.64±18.14^*^ | 33.48±23.11 |
| BmuPB008332, histone H2B | 36.31±7.63 | 20.71±7.56^*^ | 28.39±4.59 | 27.63±12.01 |
| BmuPB019079, histone H3 | 14.99±5.87 | 8.47±4.26 | 12.06±2.97^#^ | 6.42±2.99 |
| BmuPB008324, histone H3 | 11.13±8.25 | 4.08±1.08 | 9.88±1.39^#^ | 6.42±2.16 |
| BmuPB019077, histone H3 | 8.38±2.98 | 5.12±2.87 | 4.90±0.77 | 6.40±1.30 |
| BmuPB019078, histone H4 | 112.64±4.28 | 54.51±20.75^*^ | 69.56±20.61^*^ | 61.44±21.40 |
| BmuPB012417, histone H4 | 1.01±1.74 | 0.51±0.70^#^ | 0.08±0.14^#^ | 2.07±1.03 |
| BmuPB005297, histone H2B | 11.59±2.19 | 13.49±6.49 | 5.58±0.26^*#^ | 17.20±5.39 |
| BmuPB013384, cyclin-dependent kinase 1 | 2.24±0.42 | 1.14±0.72^*^ | 1.32±0.16^*^ | 1.58±0.42 |
| BmuPB018681, cyclin A | 1.02±0.35 | 0.95±0.28^#^ | 1.10±0.25^#^ | 0.57±0.12 |
| BmuPB007490, cyclin-dependent kinase regulatory subunit | 1.69±0.67 | 1.86±0.54^#^ | 1.14±0.73 | 0.80±0.45 |
| BmuPB018166, mitotic checkpoint serine/threonine-protein kinase | 0.60±0.19 | 0.26±0.11^*^ | 0.36±0.11 | 0.35±0.13 |
| BmuPB012491, transcription factor E2F2 | 0.26±0.26 | 0.21±0.06^#^ | 0.14±0.17 | 0.08±0.03 |
| BmuPB006948, condensin complex subunit 3 | 1.43±0.28 | 0.85±0.12^*^ | 1.20±0.11 | 1.11±0.37 |
| Immune function-related genes | | | | |
| BmuPB017207, interleukin 1 beta | 3.81±1.33 | 1.15±0.13^*^ | 1.87±0.30^*^ | 0.99±0.83 |
| BmuPB017519, interleukin 10 | 0.21±0.26 | 0.24±0.16 | 0.29±0.15^#^ | 0.06±0.05 |
| BmuPB004416, B-lymphocyte antigen CD19 | 0.30±0.11 | 0.28±0.17 | 0.42±0.03^#^ | 0.24±0.09 |
| BmuPB016862, CD79A antigen | 0.27±0.12 | 0.21±0.16 | 0.52±0.20^#^ | 0.10±0.07 |
| BmuPB011881, CD79B antigen | 0.41±0.17 | 0.44±0.10^#^ | 0.41±0.24 | 0.24±0.08 |
| BmuPB017503, complement factor H | 2.05±0.99 | 0.34±0.16^*^ | 0.22±0.04^*#^ | 0.67±0.30 |
| BmuPB005626, indoleamine 2,3-dioxygenase | 26.24±14.46 | 16.16±2.43 | 21.47±1.88^#^ | 14.41±2.59 |
| BmuPB017991, 2'-5'-oligoadenylate synthetase | 1.19±0.94 | 0.86±0.44^#^ | 0.81±0.34^#^ | 0.24±0.12 |
| BmuPB020946, interferon regulatory factor 7 | 6.89±5.04 | 5.03±1.18^#^ | 5.49±0.47^#^ | 2.84±0.44 |
| BmuPB009858, interferon regulatory factor 1 | 26.89±10.99 | 18.08±3.46 | 23.64±2.79^#^ | 16.00±3.85 |
| BmuPB008081, interferon-induced GTP-binding protein | 12.79±9.86 | 5.85±0.14^#^ | 5.71±1.92 | 3.57±0.98 |
| BmuPB015356, transferrin receptor | 358.71±52.28 | 241.32±54.65^*#^ | 248.17±53.55^*#^ | 93.12±38.34 |
| BmuPB021220, lymphotoxin beta | 4.38±1.40 | 4.30±1.51 | 3.74±0.35^#^ | 2.86±0.18 |
| BmuPB000610, Z-DNA binding protein 1 | 2.36±1.89 | 1.57±0.15 | 2.24±0.63^#^ | 1.09±0.55 |

Student’s t-tests were conducted to determine significant differences between age groups. * represents significant difference compared to 6-month group (P < 0.05). # represents significant difference compared to 90-month group (P < 0.05).

**Table S5. Significantly enriched KEGG pathways in comparisons between gluteus tissues of yaks at different ages.**

| ID | Gene Ratio | Adjust P | Q value | |
| --- | --- | --- | --- | --- |
| Comparisons between 6-month and 30/60-month old | | | |  |
| ko04640 Hematopoietic cell lineage | 8/109 | 0.005 | 0.005 | |
| ko04024 cAMP signaling pathway | 12/109 | 0.007 | 0.007 | |
| ko04610 Complement and coagulation cascades | 6/109 | 0.026 | 0.025 | |
| ko04976 Bile secretion | 7/109 | 0.026 | 0.025 | |
| Comparisons between 30/60-month and 90-month old | | | |  |
| ko04640 Hematopoietic cell lineage | 13/175 | 0.000 | 0.000 | |
| ko04650 Natural killer cell mediated cytotoxicity | 16/175 | 0.000 | 0.000 | |
| ko04514 Cell adhesion molecules (CAMs) | 17/175 | 0.000 | 0.000 | |
| ko04612 Antigen processing and presentation | 13/175 | 0.000 | 0.000 | |
| ko04974 Protein digestion and absorption | 11/107 | 0.000 | 0.000 | |
| ko04062 Chemokine signaling pathway | 15/175 | 0.000 | 0.000 | |
| ko04672 Intestinal immune network for IgA production | 10/175 | 0.000 | 0.000 | |
| ko04060 Cytokine-cytokine receptor interaction | 18/175 | 0.000 | 0.000 | |
| ko05340 Primary immunodeficiency | 7/175 | 0.001 | 0.001 | |
| ko05320 Autoimmune thyroid disease | 10/175 | 0.001 | 0.001 | |
| ko04940 Type I diabetes mellitus | 8/175 | 0.005 | 0.005 | |
| ko04512 ECM-receptor interaction | 8/175 | 0.006 | 0.005 | |
| ko05321 Inflammatory bowel disease (IBD) | 8/175 | 0.008 | 0.006 | |
| ko05202 Transcriptional misregulation in cancer | 13/175 | 0.009 | 0.007 | |
| ko04660 T cell receptor signaling pathway | 9/175 | 0.020 | 0.017 | |
| ko05310 Asthma | 6/175 | 0.031 | 0.026 | |
| ko04145 Phagosome | 10/175 | 0.031 | 0.026 | |
| ko04666 Fc gamma R-mediated phagocytosis | 7/175 | 0.036 | 0.031 | |
| ko03320 PPAR signaling pathway | 6/175 | 0.039 | 0.034 | |
| ko04933 AGE-RAGE signaling pathway in diabetic complications | 7/175 | 0.042 | 0.036 | |
| ko05330 Allograft rejection | 10/175 | 0.000 | 0.000 | |
| ko05416 Viral myocarditis | 11/175 | 0.000 | 0.000 | |
| ko05332 Graft-versus-host disease | 9/175 | 0.000 | 0.000 | |
| ko05150 Staphylococcus aureus infection | 10/175 | 0.001 | 0.000 | |
| ko05166 HTLV-I infection | 14/175 | 0.047 | 0.040 | |
| ko05322 Systemic lupus erythematosus | 11/175 | 0.049 | 0.042 | |

**Table S6. FPKM values of differentially expressed genes in yak gluteus at different ages (mean ± SD).**

| ID and name of unigene | 6-month | 30-month | 60-month | 90-month |
| --- | --- | --- | --- | --- |
| Immune function-related genes | | | | |
| BmuPB005339, T-cell surface glycoprotein CD3 delta chain | 32.99±7.58 | 25.26±5.60^#^ | 26.27±6.10^#^ | 36.77±3.37 |
| BmuPB005340, T-cell surface glycoprotein CD3 gamma chain | 46.45±9.52 | 31.23±6.53^*#^ | 32.59±9.27^#^ | 46.90±2.49 |
| BmuPB005549, macrophage scavenger receptor 1 | 136.82±5.03 | 88.00±28.60^*^ | 104.57±5.40^*^ | 152.36±52.90 |
| BmuPB012636, lymphocyte cell-specific protein tyrosine kinase | 20.08±3.68 | 13.92±4.55^#^ | 14.46±2.13^*#^ | 19.87±1.06 |
| BmuPB013060, CD48 antigen | 159.62±6.17 | 80.46±13.65^*^ | 83.72±10.95^*^ | 107.36±28.55 |
| BmuPB015397, CD86 antigen | 82.55±5.23 | 49.90±19.60^*^ | 56.14±3.00^*^ | 62.39±18.74 |
| BmuPB011166, T-cell receptor beta chain V region | 28.90±4.70 | 22.41±4.83^#^ | 19.88±2.89^*#^ | 29.19±1.24 |
| BmuPB011165, T-cell receptor beta chain V region | 20.17±2.73 | 13.54±3.18^*^ | 14.11±0.84^*#^ | 18.38±2.42 |
| BmuPB015276, major histocompatibility complex, class II | 5.13±1.06 | 4.03±0.85 | 3.00±0.92^*#^ | 5.17±0.98 |
| BmuPB011767, novel protein kinase C delta type | 27.70±3.06 | 19.32±5.68^*^ | 24.05±2.07 | 24.20±4.91 |
| BmuPB021190, complement component 2 | 14.65±4.67 | 8.06±1.29^*#^ | 8.30±2.56 | 19.48±8.80 |
| BmuPB017503, complement factor H | 3.39±2.11 | 0.59±0.42^*^ | 0.24±0.14^*^ | 0.68±0.39 |
| BmuPB014902, interferon, gamma-inducible protein 30 | 75.45±13.43 | 34.30±13.77^*#^ | 43.10±3.70^*#^ | 61.13±13.95 |
| BmuPB004405, interleukin 21 receptor | 1.93±0.30 | 1.47±0.52 | 1.14±0.36^*^ | 2.15±1.23 |
| BmuPB014905, interleukin 12 receptor beta-1 | 3.77±0.91 | 2.67±0.83 | 2.24±0.55^*^ | 3.39±0.81 |
| BmuPB020673, interleukin 2 receptor beta | 17.05±2.45 | 9.81±2.86^*^ | 12.12±2.86^*^ | 13.99±3.10 |
| BmuPB018203, interleukin 2 receptor gamma | 38.99±3.54 | 26.77±1.77^*#^ | 25.46±4.24^*^ | 30.22±2.18 |
| Genes related to muscle injury and regeneration | | | | |
| BmuPB017342, collagen, type III, alpha | 187.83±79.84 | 114.42±26.57^#^ | 134.87±19.54^#^ | 70.11±15.15 |
| BmuPB017681, collagen, type VI, alpha | 47.72±9.13 | 45.70±13.80^#^ | 41.47±14.54 | 25.02±4.06 |
| BmuPB004551, elastin | 56.70±19.77 | 46.11±7.76^#^ | 43.36±4.84^#^ | 25.64±6.59 |
| BmuPB018955, collagen, type V/XI/XXIV/XXVII, alpha | 10.57±1.82 | 9.99±1.63^#^ | 9.43±4.39 | 4.98±0.65 |
| BmuPB003309, collagen, type I, alpha | 33.26±14.67 | 14.03±4.61^*^ | 21.37±12.94 | 7.63±4.63 |
| BmuPB007908, collagen, type I, alpha | 61.58±22.86 | 38.58±9.69 | 49.92±11.18^#^ | 24.84±9.89 |
| BmuPB011599, C-C chemokine receptor type 1 | 5.64±0.80 | 3.53±1.25^*#^ | 5.07±1.72 | 5.44±0.58 |
| BmuPB003417, C-C chemokine receptor type 7 | 2.99±0.56 | 1.75±0.10^*^ | 1.37±0.50^*^ | 1.89±0.60 |
| BmuPB002860, C-C motif chemokine 4 | 54.83±22.40 | 25.84±9.60^#^ | 37.32±10.89 | 42.81±7.19 |
| BmuPB011602, C-C chemokine receptor type 5 | 20.66±4.34 | 12.19±2.83^*#^ | 15.74±1.53^#^ | 22.16±0.44 |
| BmuPB011598, C-C chemokine receptor type 1 | 54.16±9.96 | 33.05±7.12^*#^ | 45.57±16.24 | 52.64±10.34 |
| BmuPB002865, C-C motif chemokine 15/23 | 140.51±18.25 | 92.86±13.79^*^ | 108.90±36.49 | 134.77±42.45 |
| BmuPB015933, tumor necrosis factor receptor superfamily member 19 | 8.33±0.84 | 4.61±1.86^*^ | 6.29±0.79^*^ | 5.94±2.86 |
| BmuPB014521, tumor necrosis factor ligand superfamily member 8 | 4.11±0.60 | 3.18±0.13^*^ | 3.40±0.48 | 4.34±0.97 |
| BmuPB018032, tumor necrosis factor ligand superfamily member 5 | 7.26±0.87 | 4.56±1.66^*^ | 3.47±1.25^*^ | 4.59±0.49 |
| BmuPB014660, tumor necrosis factor receptor superfamily member 5 | 13.60±2.64 | 7.70±1.79^*^ | 6.76±1.34^*#^ | 9.54±0.89 |
| BmuPB019234, integrin alpha 4 | 42.05±3.71 | 31.69±3.67^*^ | 28.03±3.24^*^ | 32.50±6.91 |
| BmuPB004501, integrin alpha M | 26.50±5.38 | 16.14±4.34^*#^ | 23.24±3.10 | 31.37±7.11 |
| BmuPB010730, integrin beta 7 | 44.82±5.15 | 28.21±7.84^*^ | 33.03±2.47^*^ | 37.77±4.02 |
| BmuPB009995, guanine nucleotide exchange factor VAV | 16.52±2.01 | 9.77±4.09^*^ | 11.97±0.42^*^ | 14.49±2.56 |
| BmuPB020669, Ras-related C3 botulinum toxin substrate 2 | 51.09±8.98 | 33.34±7.87^*^ | 34.05±4.42^*#^ | 44.07±4.79 |

Student’s t-tests were conducted to find significant difference between 6-month or 90 month and 30-month or 60-month. * means significantly different from 6-month group (P < 0.05). # means significantly different from 90-month group (P < 0.05).
